# Case Report: Bartholin’s gland leiomyoma masquerading as a bartholin’s cyst: a rare clinical entity and diagnostic challenges

**DOI:** 10.3389/frph.2026.1810693

**Published:** 2026-06-11

**Authors:** Oadi N. Shrateh, Peter SzontaghKishazi, Nor Azlia Abdul Wahab

**Affiliations:** 1 Department of Obstetrics and Gynecology, Our Lady of Lourdes Hospital, Drogheda, Ireland; 2 Department of Histopathology, Our Lady of Lourdes Hospital, Drogheda, Ireland

**Keywords:** bartholin cyst mimic, bartholin gland, histopathology, vulvar leiomyoma, vulvar mass

## Abstract

**Background:**

Leiomyomas are benign smooth muscle tumors that most commonly arise in the uterus but may occasionally occur in extra-uterine locations, including the vulva. Vulvar leiomyomas, especially those originating from the Bartholin gland, are uncommon and often misdiagnosed as Bartholin's cysts or abscesses, leading to diagnostic challenges.

**Case presentation:**

We report the case of a 44-year-old female with a longstanding, slowly enlarging right vulvar mass initially presumed to be a Bartholin's cyst. The patient had no significant past medical history, no allergies, no family history of malignancy, and a BMI of 24. On examination, a firm, non-fluctuant 4 × 4 cm mass was palpated in the right Bartholin's gland region. Surgical excision was performed under general anesthesia. Histopathological evaluation revealed a well-circumscribed spindle-cell tumor with desmin positivity, confirming a benign leiomyoma. There was no evidence of necrosis, atypia, or malignancy.

**Discussion:**

Vulvar leiomyomas are uncommon and can arise from smooth muscle elements such as blood vessels, the round ligament, erector pili muscles, or Bartholin's gland erectile tissue. Clinically, they present as painless, slow-growing masses and are frequently misdiagnosed preoperatively. Definitive diagnosis requires histopathology and immunohistochemistry to differentiate from leiomyosarcoma and other vulvar masses. Complete surgical excision with clear margins is the treatment of choice, with a low risk of recurrence.

**Conclusion:**

Bartholin's gland leiomyomas are uncommon benign tumors that may mimic more frequent vulvar lesions. Accurate recognition, histopathologic confirmation, and complete excision are essential for optimal management and favorable outcomes. Increased awareness of this uncommon entity can reduce misdiagnosis and improve patient care.

## Introduction

Leiomyomas are benign smooth muscle neoplasms that most commonly arise in the uterus; however, they can occur in any site containing smooth muscle cells (1). Extra-uterine leiomyomas are uncommon, and their occurrence in the vulvar region is exceptionally uncommon (2). Within the vulva, leiomyomas may arise from smooth muscle elements such as blood vessel walls, the round ligament, arrector pili muscles, or erectile tissue, including that found in the Bartholin's gland (3, 4).

Clinically, vulvar leiomyomas typically present as painless, well-circumscribed, solitary masses that may mimic more common lesions such as Bartholin's gland cysts or abscesses (5). Because of their rarity and non-specific presentation, preoperative diagnosis is often challenging, leading to frequent misdiagnosis (6) or delayed diagnosis.

Histopathological and immunohistochemical examination remains essential to confirm the diagnosis and to differentiate benign vulvar leiomyomas from malignant smooth muscle tumors such as leiomyosarcoma (7). Here, we report a case of leiomyoma arising in the Bartholin's gland region, emphasizing the diagnostic challenges, histopathologic characteristics, and management approach, thereby contributing to the limited literature on this uncommon entity. This case highlights the diagnostic challenge posed by vulvar leiomyomas that may clinically mimic Bartholin's gland cysts. It underscores the importance of considering solid neoplasms in the differential diagnosis of persistent vulvar masses and emphasizes the role of histopathological examination in establishing a definitive diagnosis.

## Case presentation

A 44-year-old multiparous woman (P2 + 0) presented to her general practitioner in late 2024 with a complaint of a right-sided vulval swelling that she had first noticed seven years earlier, shortly after her second delivery. The lesion had gradually increased in size over the years but remained painless and did not cause discomfort during sitting, walking, or sexual intercourse. She denied any vaginal discharge, bleeding, or systemic symptoms such as fever, weight loss, or night sweats.

Her past medical history was unremarkable, with no chronic illnesses, no known drug allergies, and no family history of cancer. She was a non-smoker, and her body mass index (BMI) was 24 kg/m^2^. Her menstrual cycles were regular, and she was not using hormonal contraception at the time of presentation.

On clinical examination at her first presentation, there was a firm, non-tender, non-fluctuant swelling measuring approximately 4 × 4 cm located in the right Bartholin's gland area. The overlying skin and mucosa appeared normal, and there was no evidence of erythema, ulceration, or inguinal lymphadenopathy. Speculum examination revealed a normal cervix and vagina, and a cervical smear was performed as she was overdue for screening. Laboratory investigations revealed hemoglobin level of 13.5 g/dL, white blood cells of 8.9 × 10^9^ along with normal inflammatory markers. the patient was vitally stable and temperature readings were within normal limits.

The initial diagnosis was consistent with a right Bartholin's gland cyst, and she underwent word catheter insertion under local anesthesia after minimal drainage of clear fluid. She was advised on local care and scheduled for review after four weeks but did not attend follow-up, reporting later that the swelling had resolved spontaneously.

Several months later, the patient re-presented with recurrence of the vulval swelling, which she reported had never completely resolved. On examination, the swelling persisted in the right Bartholin's gland region, again measuring approximately 4 × 4 cm, firm and non-fluctuant in nature. There was no erythema or tenderness on palpation. Local infiltration of lignocaine was administered at the mucocutaneous junction in the region of the Bartholin's gland, after which a vertical incision was performed. However, only minimal fluid was aspirated, and multiple solid nodular areas were palpated within the lesion, raising suspicion of possible leiomyoma which would need more extensive procedure. The incision was then closed with absorbable sutures.

Given the unusual solid consistency and poor drainage, the patient was scheduled for exploration of right Bartholin's gland and excision of the mass under general anesthesia. The excision procedure was performed uneventfully. The lobulated solid mass was enucleated easily, preserving the gland with minimal blood loss. The lesion was completely excised, and the surgical margins were free of tumor. The specimen was sent for histopathological examination.

**Figure 1:**
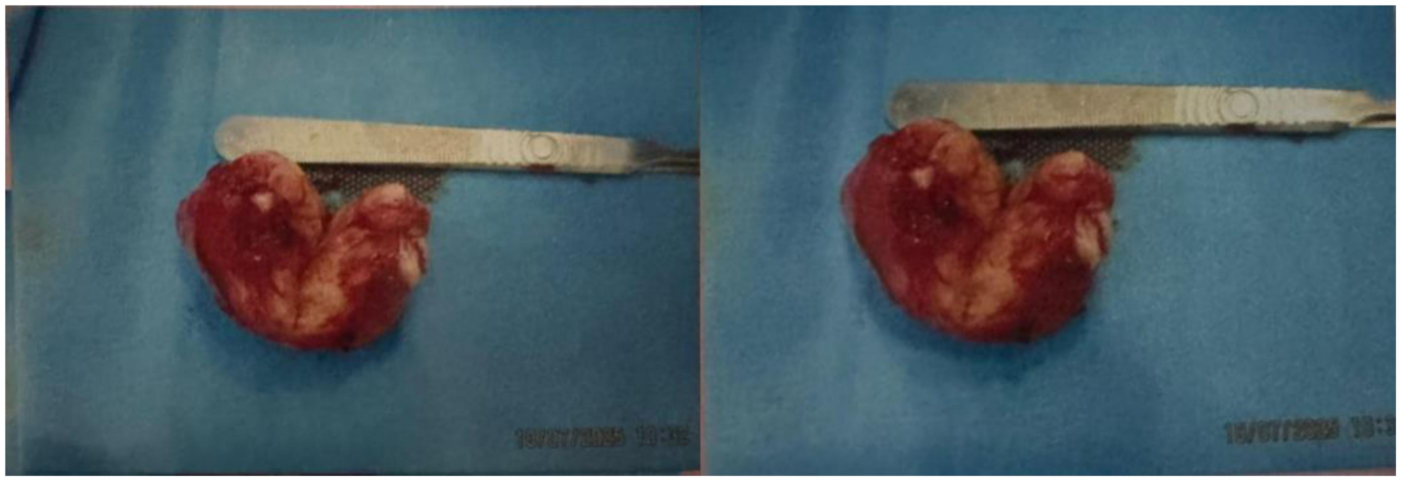
Shows the excised mass from the right bartholin’s gland.

Macroscopic examination (Figure 1) revealed a firm, brown, previously opened mass measuring 50 × 45 × 35 mm, with an area of attached squamous mucosa (13 × 10 mm). The cut surface was lobulated, whorled, and creamy-yellow, consistent with a smooth muscle lesion.

**Figure 2:**
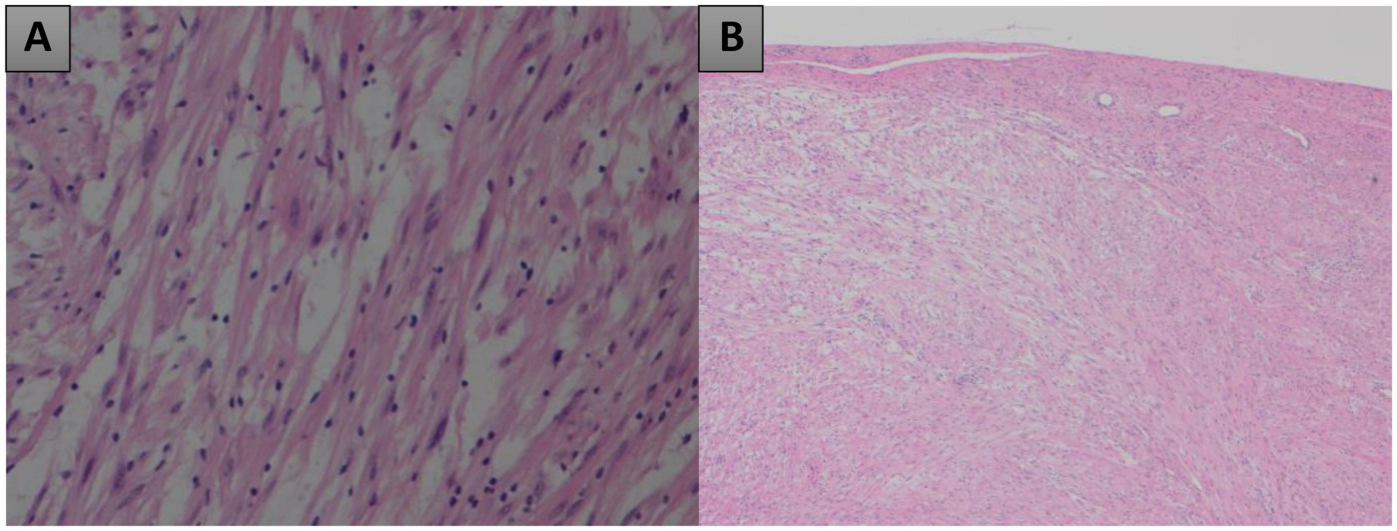
**(A)** High-power view (H&E, × 20) demonstrating uniform spindle-shaped smooth muscle cells with eosinophilic cytoplasm and elongated blunt-ended nuclei, without cytological atypia, necrosis, or increased mitotic activity. **(B)** Low-power view (H&E, × 4) showing a well-circumscribed subepithelial mesenchymal tumour composed of interlacing fascicles in a whorled pattern beneath the vulvar squamous epithelium.

Microscopic examination (Figure 2) showed a well-circumscribed tumour composed of spindle-shaped smooth muscle cells arranged in interlacing fascicles beneath focally ulcerated vulval mucosa. Immunohistochemical staining demonstrated positivity for desmin and negativity for S100, supporting smooth muscle differentiation and excluding neural tumors. Additional markers such as smooth muscle actin (SMA), h-caldesmon, or Ki-67 were not performed, as the characteristic histomorphological features together with desmin positivity were considered sufficient to establish the diagnosis of benign leiomyoma.

The postoperative course was uneventful. At follow-up, six months after surgery, the patient remained asymptomatic with no evidence of recurrence, and the surgical site had healed completely.

## Discussion

Leiomyomas are benign smooth muscle neoplasms that may arise in various locations outside the uterus, yet their occurrence in the vulva—particularly within the Bartholin's gland region—remains exceedingly uncommon (8). The rarity of this presentation contributes to diagnostic challenges, as these tumors are frequently mistaken for more common Bartholin's gland pathologies such as cysts or abscesses (9). In our case, the patient presented with a firm, non-fluctuant 4 × 4 cm mass in the right Bartholin's area, initially presumed to represent a Bartholin cyst, aligning with the presentation described in multiple previous reports (10, 11).

Clinically, vulvar leiomyomas tend to present as slowly enlarging, painless, well-circumscribed nodules. However, when the lesion becomes large or secondarily infected, patients may experience discomfort or dyspareunia (12). In addition, clinical features such as a firm, non-fluctuant consistency should raise suspicion for a solid lesion rather than a cystic process. In our case, this finding played a key role in prompting further diagnostic evaluation and definitive surgical management. The mean age of presentation reported in the literature is between 30 and 50 years, corresponding to the reproductive period when estrogen influence is believed to play a role in tumor growth (13). Immunohistochemical analysis typically reveals positivity for smooth muscle actin (SMA), desmin, and h-caldesmon, confirming smooth muscle origin, while Ki-67 labeling index remains low, supporting a benign nature (14).

Importantly, malignant entities such as leiomyosarcoma have been reported in lesions initially presumed to be Bartholin's cysts. This underscores the importance of maintaining a high index of suspicion and ensuring histopathological evaluation, particularly in atypical, recurrent, or solid masses (15, 16). The differential diagnosis of a vulvar mass in this location includes Bartholin's cyst or abscess, aggressive angiomyxoma, fibroepithelial stromal polyp, and leiomyosarcoma (15). Distinguishing benign leiomyomas from leiomyosarcomas is crucial, as both can present as solitary vulvar masses with overlapping histological features. Tavassoli and Norris (16) proposed histologic criteria to define malignancy, including tumor size >5 cm, infiltrative margins, >5 mitoses per 10 high-power fields, and moderate-to-severe cytologic atypia criterion was added by Nielsen et al. (9). Nucci (15) identified coagulative necrosis as an indicator of malignancy; when present alongside any of the previously mentioned features, it should raise suspicion for sarcoma. Our patient's lesion was well-circumscribed, with no evidence of atypia, mitotic activity or necrosis. These features support the benign diagnosis. Imaging was not performed initially as the lesion was clinically presumed to be a Bartholin's gland cyst based on examination findings. However, during the procedure only minimal fluid was obtained and a firm solid component was palpated, raising suspicion of an alternative diagnosis. This prompted definitive surgical exploration and excision of the mass. In retrospect, preoperative imaging such as ultrasound or magnetic resonance imaging might have assisted in further characterization of the lesion.

Surgical excision with clear margins remains the gold-standard treatment and is generally curative (17). Recurrence is rare but has been documented, especially in cases with incomplete excision or histological uncertainty (18). In the present case, complete excision was achieved under general anesthesia, and postoperative follow-up showed no recurrence, consistent with the outcomes reported in previous studies (19). Unlike uterine leiomyomas, adjuvant hormonal therapy or surveillance imaging is usually unnecessary for vulvar lesions unless there is suspicion of recurrence (20).

Comparatively, Bartholin's gland leiomyomas tend to be smaller and better circumscribed than their uterine counterparts, likely due to earlier clinical detection given the external location (21). However, both share similar histopathologic features and immunoprofiles. The literature review suggests fewer than 40 cases have been reported worldwide, highlighting the rarity of this entity (22). Hence, awareness among clinicians is critical to avoid misdiagnosis and ensure appropriate management. Because these lesions are uncommon and often resemble Bartholin's cysts clinically, preoperative diagnosis can be difficult and many cases are only identified following surgical excision and histopathological analysis.

In summary, vulvar leiomyomas, particularly those arising from the Bartholin's gland region, represent a diagnostic challenge due to their rarity and clinical resemblance to more common cystic lesions. Accurate preoperative diagnosis remains difficult; therefore, histopathological examination is essential. Complete surgical excision offers excellent prognosis, with very low recurrence rates when margins are clear. Further case documentation will enrich understanding of their biological behavior and optimize diagnostic accuracy.

### Patient perspective

The patient reported satisfaction with the management and outcome of the procedure. She expressed relief following complete excision of the mass, particularly after experiencing years of persistent swelling and uncertainty regarding the diagnosis. She remained asymptomatic at follow-up and was reassured by the benign nature of the condition.

## Conclusion

Vulvar leiomyomas arising from the Bartholin's gland region are uncommon and may clinically mimic more common cystic lesions. This case highlights the importance of considering solid tumors in the differential diagnosis of persistent Bartholin region masses and underscores the essential role of histopathological evaluation in establishing a definitive diagnosis. Complete surgical excision remains the treatment of choice and is associated with excellent prognosis.

## Data Availability

The original contributions presented in the study are included in the article/Supplementary Material, further inquiries can be directed to the corresponding author.
